# Nuclear estrogen receptor-α expression is an independent predictor of recurrence in male patients with pT1aN0 lung adenocarcinomas, and correlates with regulatory T-cell infiltration

**DOI:** 10.18632/oncotarget.4752

**Published:** 2015-07-29

**Authors:** Kyuichi Kadota, Takashi Eguchi, Jonathan Villena-Vargas, Kaitlin M. Woo, Camelia S. Sima, David R. Jones, William D. Travis, Prasad S. Adusumilli

**Affiliations:** ^1^ Thoracic Service, Department of Surgery, Memorial Sloan Kettering Cancer Center, New York, NY, USA; ^2^ Department of Pathology, Memorial Sloan Kettering Cancer Center, New York, NY, USA; ^3^ Department of Epidemiology and Biostatistics, Memorial Sloan Kettering Cancer Center, New York, NY, USA; ^4^ Center for Cell Engineering, Memorial Sloan Kettering Cancer Center, New York, NY, USA; ^5^ Department of Diagnostic Pathology, Faculty of Medicine, Kagawa University, Kagawa, Japan

**Keywords:** adenocarcinoma, lung, estrogen receptor, regulatory T-cell, recurrence

## Abstract

**Background:**

Tumor biology of estrogen receptor-α (ERα) and progesterone receptor (PR) has been studied in breast cancers. However, clinical impact in lung cancer remains controversial. In our study, we investigate whether ERα and PR expression predicts disease recurrence and correlates with immunologic factors in stage I lung adenocarcinoma.

**Methods:**

We reviewed patients with pathologic stage I resected lung adenocarcinoma. Tumors were classified according to the IASLC/ATS/ERS classification. Immunostaining of ERα and PR was performed using tissue microarrays (*n* = 913). Immunostaining of CD3+ and forkhead box P3 (FoxP3)+ lymphocyte infiltration, interleukin-7 receptor (IL-7R), and IL-12Rβ2 were performed. Cumulative incidence of recurrence (CIR) analysis was used to estimate probability of recurrence.

**Results:**

Nuclear ERα expression was observed in 157 (17%) patients and presented more frequently in females (*P* = 0.038) and smaller tumors (*P* = 0.019). Nuclear ERα expression was not identified in mucinous tumors. In pT1a patients, 5-year CIR of patients with ERα-positive tumors was significantly higher (5-year CIR, 20%) than those with ERα-negative tumors (8%; *P* = 0.018). This difference was statistically significant in males (*P* = 0.003) but not females (*P* = 0.55). On multivariate analysis, nuclear ERα expression was an independent predictor of recurrence (hazard ratio = 2.27; *P* = 0.030). In pT1a patients, nuclear ERα expression positively correlated with tumoral FoxP3+ lymphocytes (*P* < 0.001), FoxP3/CD3 index (*P* < 0.001), and IL-7R (*P* = 0.022).

**Conclusions:**

Nuclear ERα expression is an independent predictor of recurrence in pT1a lung adenocarcinomas and correlates with poor prognostic immune microenvironments.

## INTRODUCTION

Lung cancer is the leading cause of cancer-related death and its incidence is increasing in women [1]. Gender has been shown to play a prognostic role in lung cancer. Compared to men, the proportions of adenocarcinoma histology and early-stage disease are greater in women with lung cancers [2, 3]. Women have a higher response rate to chemotherapy and a better survival rate than men, especially in adenocarcinomas [3, 4]. In addition, mutations in *epidermal growth factor receptor* (*EGFR*), which can predict sensitivity to *EGFR* tyrosine kinase inhibitor, are more frequently identified in women than in men [5, 6]. This suggests that lung cancer carcinogenesis should be considered, at least partly, as a distinct entity by gender.

The tumor biology of sex steroid hormone receptors, such as the estrogen receptor (ER) and the progesterone receptor (PR), has been studied, especially in breast cancers [7–12]. In human ERs, there are two isoforms (ERα and ERβ) with partial homology, yet distinct function, in normal and neoplastic cells [13]. In breast cancer patients, nuclear expression of ERα and PR has been an important and favorable prognostic biomarker with a greater response to endocrine therapy (such as tamoxifen) [7–9]. Currently, immunohistochemical assessment of ERα and PR has been part of routine clinical practice for treating breast cancers. In addition to ERα, since the discovery of a second ER—which has been identified as ERβ—its functional and prognostic importance has been also investigated in breast cancers [10–12]. Recently, in lung cancers the positive association between ER expression and *EGFR* mutations has been detected [14, 15], and the potential clinical impact of ERα, ERβ, and PR has also been investigated [14–23]. Despite these investigations, their prognostic value remains controversial.

The tumor immune microenvironment has a prognostic impact on solid malignancies [24–26]. Using a large cohort of stage I lung adenocarcinoma patients, we have identified forkhead box P3 (FoxP3)+/CD3+ lymphocytes infiltration index—which represents the ratio of regulatory T cells to total T cells—in tumor-related stroma, overexpression of tumoral interleukin-7 receptor (IL-7R), and loss of tumoral IL-12Rβ2 as independent prognostic factors [27]. In breast carcinomas, associations between the tumor immune microenvironment and ER status have been investigated; the number of tumor-infiltrating lymphocytes (including FoxP3+, CD8+ or CD20+ cells) is greater in ERα-negative tumors than in ERα-positive tumors [28–31] and lymphocyte infiltration contributes to better clinical outcomes in ERα-negative tumors than in ERα-positive tumors [32].

In 2011, the International Association for the Study of Lung Cancer (IASLC), the American Thoracic Society (ATS), and the European Respiratory Society (ERS) published the new lung adenocarcinoma histologic classification system [33]. Its prognostic value—which is based on predominant histologic patterns—has been confirmed in large independent cohorts worldwide [34–36]. Additionally, our group has reported molecular and radiologic correlations with histologic subtypes [37–40]. However, associations between histologic subtypes and sex steroid hormone receptors in lung adenocarcinoma have yet to be investigated.

In our study, we investigate whether ERα and PR expression predicts risk of disease recurrence and if it has any associations with clinicopathologic factors, histologic patterns, mutation status, or immune factors in stage I lung adenocarcinoma patients.

## RESULTS

### Patient demographics

Patient demographics are shown in Table 1. Of all (*n* = 913), median patient age was 69 years (range, 23–96 years). More than half of the patients were women (*n* = 564) and had stage IA disease (*n* = 636). Median tumor size was 2.0 cm (range, 0.3–5.0). During the study period, 14% (*n* = 130) of patients experienced recurrence and 17% (*n* = 136) died from any cause without documented recurrence. Median follow-up period for patients who did not experience recurrence was 38.5 months (range, 2.6–160.1 months).

**Table 1:** Patients demographics and its associations with nuclear ERα in all patients

Variables	N	Nuclear ERα, N (%)				*P*
		Negative		Positive		
Age, years						0.63
Median	69*	69*		69*		
Range	23–96**	23–96**		43–87**		
Sex						**0.038**
Female	564	455	(81)	109	(19)	
Male	349	301	(86)	48	(14)	
Smoking status						0.56
Never	151	128	(85)	23	(15)	
Former/current	762	628	(82)	134	(18)	
Surgery						0.019
Lobectomy	718	606	(84)	112	(16)	
Limited resection	195	150	(77)	45	(23)	
Tumor size (cm)						**0.006**
Median	2.0*	2.0*		1.8*		
Range	0.3–5.0**	0.3–5.0**		0.5–5.0**		
Pathological stage						0.98
IA	636	526	(83)	110	(17)	
IB	277	230	(83)	47	(17)	
Architectural grade						0.37
Low	111	93	(84)	18	(16)	
Intermediate	579	472	(82)	107	(18)	
High	223	191	(86)	32	(14)	
Pleural invasion						0.26
Absence	758	633	(84)	125	(16)	
Presence	155	123	(79)	32	(21)	
Lymphatic invasion						0.39
Absence	622	510	(82)	112	(18)	
Presence	291	246	(85)	45	(15)	
Vascular invasion						0.45
Absence	679	558	(82)	121	(18)	
Presence	234	198	(85)	36	(15)	
Necrosis						0.75
Absence	761	632	(83)	129	(17)	
Presence	152	124	(82)	28	(18)	
Nuclear atypia						0.47
Mild	392	331	(84)	61	(16)	
Moderate	315	255	(81)	60	(19)	
Severe	206	170	(83)	36	(17)	
Mitotic count						0.98
Low	442	367	(83)	75	(17)	
Intermediate	196	162	(83)	34	(17)	
High	275	227	(83)	48	(17)	

Significant *P-*values (<0.05) are shown in bold.

ER, estrogen receptor

* Median

** Range

### ER and PR expression profiles

**Figure 1:**
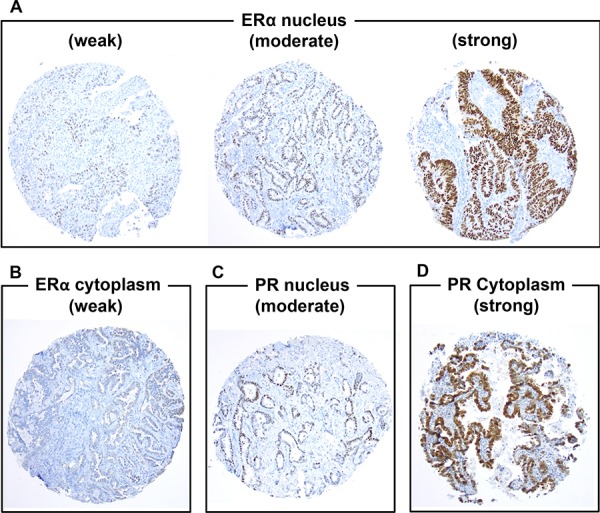
Immunohistochemical analyses of estrogen receptor-α (ERα) and progesterone receptor (PR) using tissue microarrays (original magnification, A–F: × 100 magnification) **A.** ERα is weakly, moderately, or strongly positive in tumor nuclei. **B.** ERα is weakly positive in tumor cytoplasm. **C.** PR is moderately positive in tumor nuclei. **D.** PR is strongly positive in tumor cytoplasm.

ERα and PR expression profiles are summarized in Table 2. Of all, nuclear ERα expression was observed in 157 (17%) patients, most of whom were focally positive (*n* = 138; 88%). Among nuclear ERα-positive tumors, 118 (75%) were weakly positive, 27 (17%) were moderately positive, and 12 (8%) were strongly positive (Fig. 1A). Cytoplasmic ERα expression was observed in 86 (9%) patients, most of whom were weakly (*n* = 80; 93%) (Fig. 1B) and focally positive (*n* = 74; 86%). Nuclear PR expression was observed in 119 (13%) patients, most of whom were focally positive (*n* = 108; 91%). Among nuclear PR-positive tumors, 93 (78%) were weakly positive, 20 (17%) were moderately positive (Fig. 1C), and 6 (5%) were strongly positive. Cytoplasmic PR expression was observed in 116 (13%) patients, more than half of whom were focally positive (*n* = 80; 69%). Among cytoplasmic PR-positive tumors, 87 (75%) were weakly positive, 21 (18%) were moderately positive, and 8 (7%) were strongly positive (Fig. 1D).

**Table 2:** ERα and PR expression profiles in all patients

			Intensity						Distribution			
Marker	Positive		Weak		Moderate		Strong		Focal		Diffuse	
	N	(%)	N	(%)	N	(%)	N	(%)	N	(%)	N	(%)
**ERα (n = 913)**												
Nucleus	157	(17)	118	(75)	27	(17)	12	(8)	138	(88)	19	(12)
Cytoplasm	86	(9)	80	(93)	6	(7)	0	(0)	74	(86)	12	(14)
**PR (n = 910)**												
Nucleus	119	(13)	93	(78)	20	(17)	6	(5)	108	(91)	11	(9)
Cytoplasm	116	(13)	87	(75)	21	(18)	8	(7)	80	(69)	36	(31)

ER, estrogen receptor; PR, progesterone receptor

### ERα and PR expression associations with disease recurrence and overall survival

**Figure 2:**
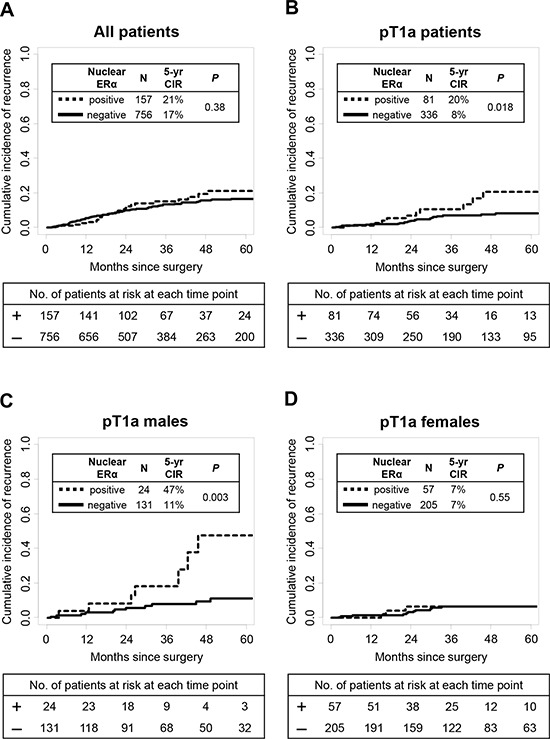
Estrogen receptor-α (ERα) associations with disease recurrence **A.** In all patients, nuclear ERα expression was not associated with risk of recurrence (*P* = 0.38) **B.** In pT1a patients, 5-year CIR of patients with nuclear ERα-positive tumors was significantly higher (*n* = 81; 5-year CIR, 20%) than patients with nuclear ERα-negative tumors (*n* = 336; 5-year CIR, 8%; *P* = 0.018). **C.** In pT1a males, 5-year CIR of patients with nuclear ERα-positive tumors was significantly higher (*n* = 24; 5-year CIR, 47%) than patients with nuclear ERα-negative tumors (*n* = 131; 5-year CIR, 11%; *P* = 0.003). **D.** In pT1a females, nuclear ERα expression was not associated with risk of recurrence (*P* = 0.55).

In all patients, nuclear ERα expression was not associated with risk of recurrence (*P* = 0.38) (Fig. 2A). In pT1a patients, 5-year CIR of patients with nuclear ERα-positive tumors was significantly higher (*n* = 81; 5-year CIR, 20%) than nuclear ERα-negative tumors (*n*= 336; 5-year CIR, 8%; *P* = 0.018) (Fig. 2B). Among pT1a patients, 5-year CIR of patients with nuclear ERα-positive tumors was significantly higher (*n* = 24; 5-year CIR, 47%) than nuclear ERα-negative tumors in males (*n* = 131; 5-year CIR, 11%; *P* = 0.003) (Fig. 2C) while the difference was not observed in females (*P* = 0.55) (Fig. 2D). After excluding the wedge resection group, among T1a patients who had undergone sublobar resection or lobectomy, 5-year CIR of patients with nuclear ERα-positive tumors was still higher (*n* = 60; 5-year CIR, 13%) than nuclear ERα-negative tumors (*n* = 279; 5-year CIR, 6%); the difference was not statistically significant (*P* = 0.19). Among T1a patients who had undergone lobectomy only, 5-year CIR of patients with nuclear ERα-positive tumors was slightly higher (*n* = 53; 5-year CIR, 10%) than nuclear ERα-negative tumors (*n* = 249; 5-year CIR, 5%); the difference was also not statistically significant (*P* = 0.58). Cytoplasmic ERα, nuclear PR, and cytoplasmic PR were not associated with risk of recurrence (Table 3).

**Table 3:** ERα and PR association with disease recurrence

Variables	All patients				Patients with pT1a disease			
	N	(%)	5-yr CIR	*P*	N	(%)	5-yr CIR	*P*
**ERα nucleus**				0.38				**0.018**
Negative	756	(83)	17%		336	(81)	8%	
Positive	157	(17)	21%		81	(19)	20%	
**ERα cytoplasm**				0.37				0.14
Negative	827	(91)	18%		380	(91)	11%	
Positive	86	(9)	14%		37	(9)	5%	
**PR nucleus**				0.18				0.96
Negative	791	(87)	17%		347	(83)	10%	
Positive	119	(13)	23%		69	(17)	12%	
**PR cytoplasm**				0.42				0.65
Negative	794	(87)	18%		348	(84)	10%	
Positive	116	(13)	16%		68	(16)	10%	

Significant *P*-values (<0.05) are shown in bold.

ER, estrogen receptor; PR, progesterone receptor; CIR, cumulative incidence of recurrence

Although overall survival (OS) analysis was also performed, ERα and PR expression (in nuclear or cytoplasm) were not associated with OS in all and T1a patients. In pT1a patients, limited resection (vs. lobectomy; *P* < 0.001), high architectural grade (*P* < 0.001), lymphatic invasion (*P* = 0.001), vascular invasion (*P* = 0.004), tumor necrosis (*P* = 0.043), greater nuclear atypia (*P* = 0.027), and higher mitotic count (*P* = 0.001) were significantly associated with high risk of recurrence.

On multivariate analysis of patients with pT1a disease, nuclear ERα expression remained a significant prognostic factor of increased risk of recurrence (hazard ratio [HR] = 2.27; *P* = 0.03) (Table 4A). When analyzing the effect of nuclear ERα expression on recurrence in males and females separately, nuclear ERα expression was significantly associated with a higher risk of recurrence in males (HR = 3.66; *P* = 0.009), but not in females (HR = 1.31; *P* = 0.63) (Table 4B).

**Table 4:** Multivariate analysis for disease recurrence in patients with T1a disease

(A) In patients with pT1a disease				
Variables		Hazard Ratio	95% CI	*P*
Nuclear ERα	positive vs. negative	2.27	1.08–4.77	**0.03**
Sex	males vs. females	2.19	1.06–4.53	**0.035**
Surgery	lobectomy vs. limited resection	3.55	1.69–7.49	**<0.001**
Architectural grade	high vs. intermediate	2.88	1.26–6.58	**0.012**
	high vs. low	11.67	1.60–84.75	**0.015**
Lymphatic Invasion	present vs. absent	1.18	0.56–2.51	0.67
Mitotic count	intermediate vs. low	0.67	0.23–1.95	0.46
	high vs. low	1.31	0.54–3.17	0.55

(B) Effect of nuclear ER in males and females with pT1a disease				
Variables		Hazard Ratio	95% CI	*P*
Nuclear ERα in females	positive vs. negative	1.31	0.43–4.01	0.63
Nuclear ERα in males	positive vs. negative	3.66	1.37–9.74	**0.009**
Surgery	lobectomy vs. limited resection	3.49	1.65–7.36	**0.001**
Architectural grade	high vs. intermediate	2.99	1.25–7.15	**0.014**
	high vs. low	12.55	1.80–86.96	**0.011**
Lymphatic Invasion	present vs. absent	1.12	0.53–2.4	0.77
Mitotic count	intermediate vs. low	0.69	0.23–2.02	0.49
	high vs. low	1.33	0.53–3.33	0.54

Significant *P-*values (<0.05) are shown in bold.

ER, estrogen receptor; CI, confidence interval

### Nuclear ER associations with patient clinicopathologic factors

In all patients, nuclear ERα-positive tumors were more frequently identified in females than in males (19% vs. 14%; *P* = 0.038) and they were smaller in size than nuclear ERα-negative tumors (median size, 1.8 cm vs. 2.0 cm; *P* = 0.019) (Table 1). However, these differences were not significant in pT1a tumors (*P* = 0.15 and *P* = 0.59, respectively). In all patients, nuclear ERα-positive tumors were slightly more frequently identified in the limited resection group than in the lobectomy group (23% vs. 16%; *P* = 0.019); however, this difference was not statistically significant in pT1a tumors (*P* = 0.15). The other aforementioned factors were not associated with nuclear ERα expression.

**Figure 3:**
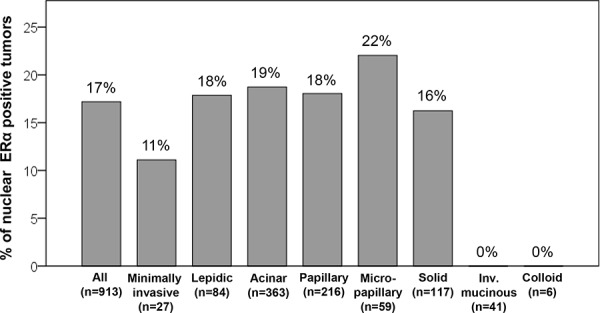
Percentage of nuclear estrogen receptor-α (ERα)-positive tumors according to histologic subtypes Nuclear ERα-positive tumors were most frequently observed in micropapillary subtype (22%), although there was no significant difference in percentages of nuclear ERα-positive tumors among non-mucinous invasive tumors (18% in lepidic subtype, 19% in acinar subtype, 18% in papillary subtype, and 16% in solid subtype). Nuclear ERα-positive tumors were not identified in mucinous subtypes (invasive mucinous or colloid subtype). Nuclear ERα-positive tumors were less frequently identified in minimally invasive subtype (11%).

According to histologic subtype, nuclear ERα-positive tumors were most frequently observed in micropapillary subtype (22%) although there was no significant difference in the rate of nuclear ERα-positive tumors among non-mucinous invasive tumors (18% in lepidic subtype, 19% in acinar subtype, 18% in papillary subtype, and 16% in solid subtype) (Fig. 3). Nuclear ERα-positive tumors were not identified in mucinous subtypes (invasive mucinous or colloid subtype). Nuclear ERα-positive tumors were less frequently identified in minimally invasive subtype (11%). Among minimally invasive tumors, two mucinous cases were negative for nuclear ERα.

Associations between nuclear ERα and mutation status were also analyzed and the results showed that nuclear ERα expression was not associated with *EGFR* or *KRAS* mutations (*P* = 0.99 and *P* = 0.6, respectively).

### Nuclear ER associations with prognostic immune markers

In all patients, nuclear ERα expression was positively associated with high tumoral and stromal CD3+ lymphocyte infiltration (*P* = 0.012 and *P* = 0.043, respectively), high tumoral and stromal FoxP3+ lymphocyte infiltration (*P* < 0.001 and *P* = 0.027, respectively), and high tumoral and stromal FoxP3/CD3 risk index (*P* < 0.001 and *P* = 0.006, respectively) (Table 5). In patients with pT1a disease, nuclear ERα expression was positively associated with high stromal CD3+ lymphocyte infiltration (*P* < 0.001), high tumoral and stromal FoxP3+ lymphocyte infiltration (*P* < 0.001 and *P* = 0.028, respectively), high tumoral FoxP3/CD3 risk index (*P* < 0.001), and high tumoral IL-7R expression (*P* = 0.022) (Table 5). In male patients with pT1a disease, nuclear ERα expression was positively associated with high tumoral CD3+ lymphocyte infiltration (*P* < 0.001), and high tumoral FoxP3/CD3 risk index (P = 0.001). However, these immune markers were not associated with smoking status or patient gender (data not shown).

**Table 5:** Associations between nuclear ERα and immune markers

Variable	(A) All patients					(B) Patients with pT1a disease				
	Nuclear ERα, N (%)				*P*	Nuclear ERα, N (%)				*P*
	Negative		Positive			Negative		Positive		
Tumoral CD3					**0.012**					0.07
Low	425	(86)	72	(14)		179	(84)	34	(16)	
High	314	(79)	84	(21)		149	(76)	46	(24)	
Stromal CD3					**0.043**					**<0.001**
Low	501	(85)	89	(15)		235	(86)	39	(14)	
High	224	(79)	59	(21)		87	(70)	37	(30)	
Tumoral FoxP3					**<0.001**					**<0.001**
Low	391	(91)	38	(9)		168	(92)	15	(8)	
High	341	(75)	116	(25)		155	(71)	63	(29)	
Stromal FoxP3					**0.027**					**0.028**
Low	471	(85)	85	(15)		223	(84)	44	(16)	
High	241	(79)	66	(21)		98	(74)	35	(26)	
Tumoral FoxP3/CD3 index					**<0.001**					**<0.001**
Low	457	(89)	56	(11)		196	(88)	26	(12)	
High	273	(74)	98	(26)		127	(71)	52	(29)	
Stromal FoxP3/CD3 index					**0.006**					0.07
Low	526	(85)	92	(15)		236	(83)	48	(17)	
High	175	(77)	53	(23)		78	(74)	27	(26)	
IL-7 R					0.41					**0.022**
Low	440	(84)	85	(16)		228	(84)	44	(16)	
High	299	(81)	68	(19)		98	(74)	35	(26)	
IL-12Rβ2					0.96					0.96
Low	557	(83)	117	(17)		238	(80)	60	(20)	
High	180	(82)	39	(18)		88	(81)	21	(19)	

Significant *P-*values (<0.05) are shown in bold.

ER, estrogen receptor; FoxP3, forkhead box P3; IL-7R, interleukin-7 receptor; IL-12Rβ2, interleukin-12 receptor β2

## DISCUSSION

We have demonstrated that nuclear ERα expression is an independent predictor of increased risk of recurrence in patients with pT1aN0 (≤2.0 cm) lung adenocarcinoma, especially in male patients, and positively correlates with poor prognostic immune microenvironments (CD3+ and FoxP3+ lymphocyte infiltration, and tumoral IL-7R expression).

In lung cancers, ER expression was reported to correlate with female sex, less smoking history, smaller tumor size, adenocarcinoma histology, and *EGFR* mutation [14, 15, 20–23]. Our results from stage I lung adenocarcinoma show that ERα expression was more frequently observed in females than males (19% vs. 14%) and correlates with smaller tumor size (1.8 cm vs. 2.0 cm); however, it was not associated with smoking history or *EGFR* mutation.

Previous studies have investigated the clinical impact of ER expression in lung cancer patients [14–23]; however, its prognostic significance remains controversial. Most studies have demonstrated that nuclear ERβ expression is associated with better prognosis in lung cancers [15–18], especially in male patients [16, 17]. However, other groups also have reported the unfavorable prognostic value of nuclear and cytoplasmic ERβ expression in lung cancers [20, 22]. With regard to ERα expression in lung cancers, Raso et al. and Kawai et al. reported that cytoplasmic ERα expression was associated with worse prognosis [18, 20], while Rouquette et al. found that it had favorable prognostic value [21]. Moreover, other studies did not identify any prognostic significance of nuclear or cytoplasmic ERα expression in lung cancers [14–16, 19, 22]. By contrast, the prognostic significance of ERα in resected early-stage lung adenocarcinoma remained unknown since previous study cohorts were either composed of only a small number of patients or were a heterogeneous population with regards to pathologic TNM stage (including early-stage and advanced-stage) and histology (including adenocarcinoma and squamous cell carcinoma). In our study using a large, uniform cohort (*n* > 900) comprised of patients with stage I lung adenocarcinoma, we have demonstrated that ERα expression is an independent risk factor of disease recurrence, especially in male patients with pT1a status (HR = 3.66).

Considering the inconsistent results of previous studies—specifically clinical correlations and the prognostic value of ER expression in lung cancers—its function may vary due to variations in patient gender (male vs. female), location of ER expression (nucleus vs. cytoplasm), ER isoforms (ERα vs. ERβ), and epitopes that each anti-ER monoclonal antibody can recognize. The two anti-ERα mouse monoclonal antibodies (ID5 and 6F11) have shown similar immunoreactivity in breast carcinomas [41] and were most frequently used in previous studies on lung carcinomas [14, 16, 19–21]. As an epitope, monoclonal antibody ID5 recognizes N-terminal of ERα while 6F11 recognizes full length of ERα [20]. More recently, anti-ERα rabid monoclonal antibody SP1—which recognizes C-terminal of ERα—has been introduced and is reported to have higher sensitivity and an 8-fold higher affinity in breast carcinomas for the detection of ERα, compared with ID5 [42]. Furthermore, in breast carcinomas, SP1 was recognized as a more reliable prognostic factor and a superior predictor of response to endocrine therapy compared with ID5 [9]. In lung adenocarcinoma, the superiority of SP1 to ID5 in the detection of tumoral ERα was also demonstrated; detection rate of ERα was significantly higher in SP1 (27%) when compared with ID5 (8%) and 6F11 (14%) [43]. In our study, we first described the unfavorable prognostic value of nuclear ERα in early-stage lung adenocarcinoma using monoclonal antibody SP1, which has high sensitivity and affinity. Use of different monoclonal antibodies in the studies may be the reason why nuclear ERα expression was not associated with smoking history or *EGFR* mutations in our study.

The IASLC/ATS/ERS lung adenocarcinoma classification system has powerful prognostic value [34–36] and shows molecular correlations (such as thyroid transcription factor-1, Ki-67, *EGFR/KRAS* mutations) [37–40]. In our study, no significant difference of ERα expression rate was observed among tumors with non-mucinous invasive subtypes. However, we found it interesting that no mucinous tumors (including mucinous minimally invasive, invasive mucinous, and colloid subtypes) showed nuclear ERα expression. Invasive mucinous adenocarcinoma (mucinous bronchioloalveolar carcinoma) has been known to correlate with *KRAS* mutation and harbor no *EGFR* mutation [44, 45]. Additionally, the recently discovered fusion gene *CD74-NRG1* presented specifically in invasive mucinous lung adenocarcinoma [46, 47]. Taking this into account, invasive mucinous adenocarcinoma can be considered a distinct entity or subtype from non-mucinous adenocarcinoma of the lung, according to its molecular profile.

Estradiol-which is the most biologically active type of estrogen-was proven to stimulate lung cancer cell growth, both *in vitro* and in mouse models [48]. In lung cancer patients, high serum estrogen levels are an unfavorable prognostic marker [49] and hormone replacement therapy may correlate with decreases survival in women [50, 51]. Tamoxifen is an antagonist of ER and causes decreases in invasion capacity and proliferation of ER-positive human lung adenocarcinoma cell lines [52, 53]. Interestingly, highly concordant co-expression of estrogen receptors and aromatase-which is an enzyme that can catalyze androgen aromatization into estrogen-has been identified in lung cancers [54]. Additionally, Niikawa et al. reported that estradiol was locally produced, mainly by aromatase, in lung cancer cells and it played an important role in the growth of ER-positive tumors in *in vitro* studies, thereby suggesting that anti-estrogen therapies (such as selective ER modulators and aromatase inhibitors) may be clinically effective in patients with ER-positive lung cancers [55]. More importantly, their study also demonstrated that estradiol concentration in lung cancer tissue was significantly higher in men than in postmenopausal women [55]. This finding may explain why ERα expression in lung adenocarcinoma has had a significant prognostic impact on males but not for females in our study.

Recently, an effect of estrogen levels on regulatory T cells was investigated and it was suggested that high levels of estrogen might induce increases of regulatory T cells in peripheral blood [56, 57]. Previously, we reported high FoxP3+/CD3+ lymphocyte ratio as an independent risk factor of recurrence in stage I lung adenocarcinoma [27]. In our current study, we demonstrated that FoxP3+ regulatory T lymphocyte infiltration was positively associated with ERα expression in lung adenocarcinoma. By contrast, in breast carcinomas the degree of tumor infiltrating FoxP3+ regulatory T lymphocytes was higher in ERα-negative tumors than in ERα-positive tumors [30, 31]. This discrepancy may be due to multiple factors including variations in primary tumor location (lung vs. breast), predominant gender and exposure of the tumor microenvironment to other immunogenic proteins.

In conclusion, our study demonstrates that nuclear ERα expression is an independent predictor of increased risk of recurrence in small (pT1aN0) stage I lung adenocarcinoma, especially in males, and correlates with unfavorable prognostic immune microenvironments (FoxP3+ regulatory T lymphocyte infiltration and tumoral IL-7R overexpression). In stage I lung adenocarcinoma, nuclear ERα expression is observed in 17% of cases using monoclonal antibody SP1, which may become a potential target for anti-estrogen therapy.

## MATERIALS AND METHODS

### Patients

The Institutional Review Board at Memorial Sloan Kettering Cancer Center (MSK) approved our retrospective study (WA0269-08). We reviewed patients with pathologic stage I, solitary lung adenocarcinomas who had undergone surgical resection at MSK between 1995 and 2009. Tumor slides and blocks from 944 patients were available for slide review and tissue microarray construction. Of the 913 patients with high quality tissue cores, 718 had undergone lobectomy and 195 had undergone limited resection (segmentectomy [*n* = 65] and wedge resection [*n* = 130]). In our cohort of patients with T1a disease, 85% of patient had undergone lymph node dissection or sampling in addition to lung resection. Lymph nodes were not examined in this study.

Clinical data were collected from our prospectively maintained database. As of March 2013, patient medical records and the database of last follow-up were reviewed and update. Disease stage was assigned using the seventh edition of the *American Joint Committee on Cancer TNM Staging Manual [58]*. Of all, 497 patients had *EGFR* and *KRAS* mutation analyses results in our dataset. Subsets of the cases in this study have been used in our previous publications [27, 37–39].

### Histologic evaluation

All available tumor slides were reviewed by two pathologists (K.K. and W.D.T.), both of whom were blinded to patient clinical outcomes, using an Olympus BX51 microscope (Olympus, Tokyo, Japan) with a standard 22-mm diameter eyepiece. Tumors demonstrating squamous morphology, such as keratinization or intracellular bridges, were excluded from this study. When the tumors exhibited a purely solid pattern without differentiated adenocarcinoma morphology (e.g., lepidic, acinar, or papillary patterns), those cases were proven to have intracellular mucin with mucin stains at time of original pathologic diagnosis. Tumors were classified according to the IASLC/ATS/ERS classification system [33] and were grouped into 3 architectural grades on the basis of histologic subtype-low (adenocarcinoma *in situ*, minimally invasive, or lepidic), intermediate (papillary or acinar), and high (micropapillary, solid, invasive mucinous, or colloid) [34, 37, 39].

Mitoses were evaluated in 50 high-power fields (HPFs) at × 400 magnification (0.237 mm^2^ field) in areas with highest mitotic activity and were counted as average number of mitotic figures per 10 HPFs. Mitotic count was classified as follows: low (0–1 mitotic figures/10 HPFs), intermediate (2–4 mitotic figures/10 HPFs), and high (≥5 mitotic figures/10 HPFs) [39]. Visceral pleural invasion, lymphovascular invasion, and tumor were also investigated.

### Immunohistochemical analysis and scoring of ERα and PR expression

We briefly deparaffinized 4 μm-thick sections from previously constructed tissue microarray blocks [27, 38]. Each case had 2–6 tumoral cores and 2–3 stromal cores (0.6 mm in diameter). Using the standard avidin-biotin-complex peroxidase technique, sections were immunostained for anti-ERα antibody (SP1, Ventana; prediluted) and anti-PR antibody (1E2, Ventana; prediluted). Of all, 913 ERα cases and 910 PR cases had adequate tumor cores available for immunohistochemical analyses. In each tumor core, intensity score (0: no expression, 1: weak, 2: moderate, and 3: strong) and distribution (%) of ERα and PR expression were evaluated separately in tumor nuclei and cytoplasm [14, 20, 27]. Average of intensity scores and positive cell distribution within cores were used as the score for each patient. Intensity scores were classified into either negative (score 0), weakly (score 0–1), moderately (score 1–2), or strongly positive (score 2–3). Distribution was classified into focally (0–49%) and diffusely (≥50%) positive. Any level of positivity (score > 0) for ERα and PR was considered positive [14, 20].

Immunohistochemical scores (high or low) of CD3+ lymphocyte infiltration (in tumor and stroma), FoxP3+ lymphocyte infiltration (in tumor and stroma), CD3/ FoxP3+ risk index (in tumor and stroma), tumoral IL-7R, and tumoral IL-12Rβ2 were obtained from the dataset used in our previous study [27].

### Statistical analysis

Associations between variables were analyzed using Fisher's exact test for categorical variables and the Wilcoxon test for continuous variables. Follow-up duration was calculated from date of surgery to date of first recurrence, death from any cause, or last follow-up. Cumulative incidence of recurrence (CIR) where death from any cause other than recurrence was considered a competing event was used to estimate probability of recurrence [59]. Differences in CIR between groups were assessed using the Gray method for univariate analyses and the Fine-Gray method for multivariate analyses, after adjustment for important potential confounders [60]. Overall survival was estimated using the Kaplan–Meier method, and nonparametric group comparisons were performed using the logrank test. All *P-*values were determined using two-tailed statistical analyses and *P* < 0.05 was considered statistically significant. Statistical analyses were conducted using SAS v9.2 (SAS Institute, Cary, NC) and R (R Development Core Team, 2010), including the “survival” and “cmprsk” packages.
